# Microwave Imaging of Anisotropic Objects by Artificial Intelligence Technology

**DOI:** 10.3390/s23218781

**Published:** 2023-10-27

**Authors:** Shu-Han Liao, Chien-Ching Chiu, Po-Hsiang Chen, Hao Jiang

**Affiliations:** 1 Department of Electrical and Computer and Engineering, Tamkang University, New Taipei City 251301, Taiwan; shliao@gms.tku.edu.tw (S.-H.L.); 810440031@gms.tku.edu.tw (P.-H.C.); 2 School of Engineering, San Francisco State University, San Francisco, CA 94117-1080, USA; jianghao@sfsu.edu

**Keywords:** microwave imaging, anisotropic objects, convolution neural network, dielectric objects, artificial intelligence, inverse scattering problem

## Abstract

In this paper, we present the microwave imaging of anisotropic objects by artificial intelligence technology. Since the biaxial anisotropic scatterers have different dielectric constant components in different transverse directions, the problems faced by transverse electronic (TE) polarization waves are more complex than those of transverse magnetic (TM) polarization waves. In other words, measured scattered field information can scarcely reconstruct microwave images due to the high nonlinearity characteristic of TE polarization. Therefore, we first use the dominant current scheme (DCS) and the back-propagation scheme (BPS) to compute the initial guess image. We then apply a trained convolution neural network (CNN) to regenerate the microwave image. Numerical results show that the CNN possesses a good generalization ability under limited training data, which could be favorable to deploy in image processing. Finally, we compare DCS and BPS reconstruction images for anisotropic objects by the CNN and prove that DCS is better than BPS. In brief, successfully reconstructing biaxial anisotropic objects with a CNN is the contribution of this proposal.

## 1. Introduction

Microwave imaging is an emerging imaging technology that uses electromagnetic waves to irradiate the unknown object. This technology eliminates the pressure and radiation problems associated with X-ray detection in the detection of breast cancer. Microwave devices are far more capable of receiving scattered field information than X-ray devices, which enables them to reconstruct high-resolution images in complex environments. Therefore, some scholars have attached great importance to medical imaging techniques in recent years, such as computed tomography and magnetic resonance imaging, that have wide applications and significant developments. There are still some problems to be investigated: poor penetration, improper positioning, nonlinearity, the curse of dimensionality, etc. In order to solve the aforementioned problems, the general algorithm for solving the inverse scattering problem (ISP) converts the ISP into an optimization problem.

In the past, the methods for solving the inverse scattering problem could be divided into two major categories: traditional algorithms [1,2,3,4,5,6,7,8,9,10,11,12,13,14,15,16,17,18,19,20,21,22,23,24,25,26,27,28,29,30,31,32,33,34,35,36,37,38,39,40] and neural networks [41,42,43,44,45,46,47,48,49,50,51,52,53,54,55,56,57,58,59,60,61,62,63,64,65,66,67]. Traditional algorithms to solve the ISP have been published in recent years. In 2021, Zhao proposed a highly efficient inversion method that utilized the improved subspace-regularized distorted-Born iterative method and the multilevel Green’s function interpolation method to solve 3D ISP [23]. In 2022, Wei proposed a subspace-based, distorted finite difference frequency domain-based iterative method to improve the efficiency of electromagnetic scattering inversion [33]. In 2023, Sun proposed cross-correlated contrast source inversion in homogeneous background media [40]. Some researchers used AI technology to solve the ISP in recent years. In 2019, Chen proposed the dominant current scheme (DCS) to calculate the initial guess image to be inputted into a convolution neural network (CNN) for reconstructing high-resolution images that had effectively converged with the training procedure [42]. In 2020, Shao proposed deep learning networks, which can reduce the difficulty of training by a two-stage training process [45]. In 2021, Zhou proposed a modified contrast scheme to solve the 2D and 3D ISP. This method could reconstruct high relative permittivity [50]. In 2022, Xu proposed end-to-end scalable cascaded convolution neural networks to solve the ISP. He inputted scattered information to the cascaded CNN blocks to reconstruct high-resolution images. The advantages of the above methods are that real-time reconstructed high-resolution images and high performance can be obtained. The microwave imaging technology has also made significant discoveries in the biomedical imaging problem [59]. In 2023, Zhang proposed a two-stage neural network approach to solve the ISP. In the first step, the single frequency scattered field information was inputted to a deep residual convolutional neural network, and the multi-frequency scattered field information was predicted through a training process. In the second step, the predicted multi-frequency scattered field information was further inputted to a deep convolutional encoder–decoder, which was used to reconstruct the exact dielectric constant distribution. In the numerical results, it was confirmed that this method could accurately reconstruct inhomogeneous and high-contrast scatterers [67].

Since biaxial anisotropic scatterers have different components of dielectric coefficients in different transverse directions, the problem is more complicated than that of pure transverse magnetic (TM) polarization. Recently, a few studies have been published on anisotropic objects [68,69,70,71,72,73]. In 1998, Chiu reconstructed a perfect biaxial conductor using an uncorrelated illumination method. Numerical results illustrated that the proposed method could reconstruct materials of large dielectric coefficient distribution. Noise interference had also been considered subsequently [68]. Ye introduced the subspace-based distorted-Born iteration method to reconstruct biaxial objects in 2020. Numerical results showed that the proposed method was capable of reconstructing high-resolution images with good noise immunity [72]. In 2022, Ye proposed to use a generative adversarial network with a visual geometry group and total variation loss function to reconstruct biaxial objects. Numerical results demonstrated that the proposed method was able to reconstruct high-resolution images rapidly [56]. Next, Chiu also proposed an AI technique to reconstruct uniaxial objects. The scattered field information obtained from our first measurement was used to estimate the preliminary permittivity distribution by back-propagation scheme (BPS) and DCS. It was then being inputted to U-Net and trained to reconstruct an accurate permittivity distribution. Numerical results elucidated that our proposed method had high performance in the reconstruction of uniaxial objects, especially in the dielectric coefficient distribution aspect [73].

The contributions of this paper are summarized as follows:

(1) We have successfully utilized neural networks and deep learning technology to reconstruct anisotropic objects in the microwave imaging research field. Compared to [74], our method does not need to designate the incident angle. It is worth mentioning that under the same noise level and training parameter settings, our proposed method can still reconstruct accurate anisotropic microwave images.
(2) We have reconstructed anisotropic objects primarily by BPS and DCS, which are more complicated, by just modifying the formula process. Transverse electronic (TE) polarized waves have x and y vectors, while TM-polarized waves are scalars. The correlated x and y field vectors may cause more difficulty to reconstruct images than with the TM wave as well as in uniaxial cases. In brief, the multiple scattering problems of TE-polarized waves is more serious than that of TM-polarized waves.
(3) As far as we are aware, using a DCS initial guess with a CNN on anisotropic objects has not yet been studied. In this paper, we successfully implemented DCS with a CNN upon anisotropic objects and acquired good reconstruction results. In addition to that, our proposed method also successfully achieves real-time imaging.
(4) Numerical results show that our proposed method is able to reconstruct different permittivity distributions efficiently. And this has been verified from the experimental data measured by the Fresnel Institute.
(5) The GAN architecture introduced by Ye et al. [56] required an extended training period, whereas the U-Net architecture developed by us delivers exceptional performance in significantly less computational time. To summarize, we can attain excellent reconstruction results by leveraging our novel selection method for anisotropic objects.

We introduce the theory and formula in Section 2. The CNN architecture is described in Section 3. In Section 4, we analyze the numerical results. Section 5 presents the conclusions.

## 2. Theoretical Formulations

### 2.1. Forward Problem

**Figure 1:**
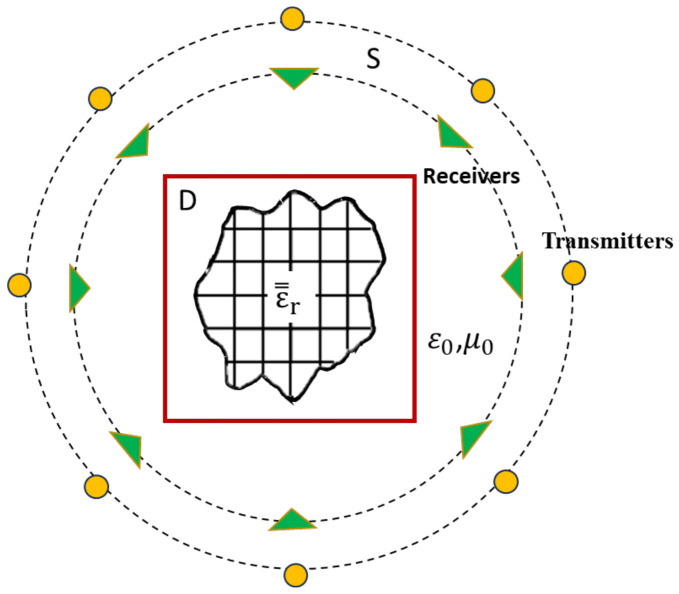
Typical schematic of the problem in the plane.

We assume that the scatterer is placed in free space with infinite extension in the Z directions, as in Figure 1. We denote the dielectric constant tensor of the scatterer as ${\overset{=}{\varepsilon }}_{r}$ and the magnetic permeability as $\mu_{0}$. The diagonal matrix of ${\overset{=}{\varepsilon }}_{r}$ is given by the $\varepsilon_{x}\;\left(x,y\right)$, $\varepsilon_{y}\;\left(x,y\right)$, and $\varepsilon_{z}\;\left(x,y\right)$ in the x, y, and z directions.

In this paper, we focus on the reconstruction of anisotropic objects in free space with $e^{j\omega t}$. Two different incident waves are considered as the following.

#### 2.1.1. Transverse Magnetic Waves

$E_{z}^{i}\left(\bar{r}\right)$ represents the incident electric field; $k_{0}$ denotes the wave number in free space; and ∅ is the incident angle.

(1) $${\bar{E}}^{i}\left(\rho \right)=E_{z}^{i}\left(\rho \right)\hat{z}{\;}^{\;}=e^{-jk_{0}(xcos\emptyset +ysin\emptyset )}\hat{z}$$

The scattered field has the Z component only since the incident wave has only the z-component, and the scatterer has infinite extension in the Z directions. The total field $\bar{E}=E_{z}\hat{z}$, and the scattered field ${\bar{E}}^{s}=E_{z}^{s}\hat{z}$ can be written as the following equations:

(2) $$E_{z}\left(\rho \right)=\int_{s}^{}G\left(\rho ,\;\rho \prime \right)\left(\varepsilon_{z}\left(\rho \prime \right)-1\right)E_{z}\left(\rho \prime \right)ds^{\prime }+E_{z}^{i}\left(\rho \right),\;\rho ,\rho \prime \in S$$

(3) $$E_{z}^{s}\left(\rho \right)=\int_{s}^{}G\left(\rho ,\rho \prime \right)\left(\varepsilon_{z}\left(\rho \prime \right)-1\right)E_{z}\left(\rho \prime \right)ds^{\prime },\;\rho \notin S,\rho \prime \in S$$

where $G\left(\rho ,\rho \prime \right)=-\frac{{\mathrm{jk}}_{0}^{2}}{4}H_{0}^{\left(2\right)}\left(k_{0}\left|\rho -\rho \prime \right|\right)$ is the two-dimensional free-space Green’s function, and $H_{0}^{\left(2\right)}$ is the zero-order Hankel function of the second kind.

#### 2.1.2. Transverse Electric Waves

The incident waves $E_{x}^{i}\left(\rho \right)$ and $E_{y}^{i}\left(\rho \right)$ can be expressed as the following:

(4) $$E_{x}^{i}\left(\rho \right)=-sin\emptyset e^{-jk_{0}\left(xcos\emptyset +ysin\emptyset \right)}$$

(5) $$E_{y}^{i}\left(\rho \right)=cos\emptyset e^{-jk_{0}\left(xcos\emptyset +ysin\emptyset \right)}$$

Due to the coupling relationship between $E_{x}$ and $E_{y}$, we employ the vector potential technique to calculate the scattered fields with the given incident field ${\bar{E}}^{i}\left(\rho \right)=E_{x}^{i}\left(\rho \right)\hat{x}+E_{y}^{i}\left(\rho \right)\hat{y}$. The total field $\bar{E}\left(\rho \right)=E_{x}\left(\rho \right)\hat{x}+E_{y}\left(\rho \right)\hat{y}$ and the external scattered field ${\bar{E}}^{s}\left(\rho \right)=E_{x}^{s}\left(\rho \right)\hat{x}+E_{y}^{s}\left(\rho \right)\hat{y}$ are expressed as the following equations:

(6) $$E_{x}\left(\rho \right)=\left(\frac{\partial^{2}}{\partial x^{2}}+k_{0}{}^{2}\right)\left[\int_{s}^{}G\left(\rho ,\rho \prime \right)\left(\varepsilon_{x}\left(\rho \prime \right)-1\right)E_{x}\left(\rho \prime \right)ds^{\prime }\right]+\frac{\partial^{2}}{\partial x\partial y}\left[\int_{s}^{}G\left(\rho ,\rho \prime \right)\left(\varepsilon_{y}\left(\rho \prime \right)-1\right)E_{y}\left(\rho \prime \right)ds^{\prime }\right]+{\vec{E}}_{x}^{i}\left(\rho \right)$$

(7) $$E_{y}\left(\rho \right)=\frac{\partial^{2}}{\partial x\partial y}\left[\int_{s}^{}G\left(\rho ,\rho \prime \right)\left(\varepsilon_{x}\left(\rho \prime \right)-1\right)E_{x}\left(\rho \prime \right)ds^{\prime }\right]+\left(\frac{\partial^{2}}{\partial x^{2}}+k_{0}{}^{2}\right)\left[\int_{s}^{}G\left(\rho ,\rho \prime \right)\left(\varepsilon_{y}\left(\rho \prime \right)-1\right)E_{y}\left(\rho \prime \right)ds^{\prime }\right]+{\vec{E}}_{y}^{i}\left(\rho \right)$$

(8) $$E_{x}^{s}\left(\rho \right)=\left(\frac{\partial^{2}}{\partial x^{2}}+k_{0}{}^{2}\right)\left[\int_{s}^{}G\left(\rho ,\rho \prime \right)\left(\varepsilon_{x}\left(\rho \prime \right)-1\right)E_{x}\left(\rho \prime \right)ds^{\prime }\right]+\frac{\partial^{2}}{\partial x\partial y}\left[\int_{s}^{}G\left(\rho ,\rho \prime \right)\left(\varepsilon_{y}\left(\rho \prime \right)-1\right)E_{y}\left(\rho \prime \right)ds^{\prime }\right]$$

(9) $$E_{y}^{s}\left(\rho \right)=\frac{\partial^{2}}{\partial x\partial y}\left[\int_{s}^{}G\left(\rho ,\rho \prime \right)\left(\varepsilon_{x}\left(\rho \prime \right)-1\right)E\hat{x}\left(\rho \prime \right)ds^{\prime }\right]+(\frac{\partial^{2}}{\partial x^{2}}+{k_{0}}^{2})\left[\int_{s}G\left(\rho ,\rho \prime \right)\left(\varepsilon_{y}\left(\rho \prime \right)-1\right)E\hat{y}\left(\rho \prime \right)ds^{\prime }\right]$$

In the TM case, we assume that the dielectric coefficient distribution is known. Then, we can find the total and scattered electric fields in the Z direction by Equations (1)–(3). Similarly, we can find the total and scattered electric fields in the X and Y directions for the TE case by using Equations (4)–(9).

### 2.2. Inverse Problem

Scattered field information was used to guess the dielectric coefficient distributions by BPS and DCS, respectively, in inverse scattering. This can effectively reduce the training process difficulty of the neural network. In the following step, the preliminary permittivity distributions of BPS and DCS are inputted to the CNN for reconstruction. The detailed formulation of BPS and DCS can be found in [73]. The trained CNN model is used to reconstruct in real time more accurate permittivity distributions.

## 3. Convolutional Neural Network

**Figure 2:**
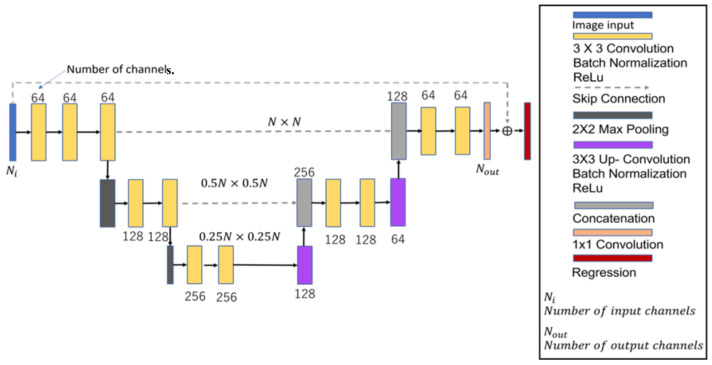
Convolution neural network architecture.

AI technology is flourishing nowadays, including vast applications in speech recognition, image processing, and self-driving vehicles. However, electromagnetic imaging techniques are also widely applied, such as in CNNs, deep convolutional neural networks [75,76], and generative adversarial networks, etc. In this study, we use a three-layer CNN architecture to solve the inverse scattering problem, which is shown in Figure 2. On the left half of the contraction network, a 3 × 3 convolutional layer, a normalization layer, a linear rectification layer, and a 2 × 2 pooling layer are repeatedly added. On the right half of the expansion network, a 3 × 3 convolutional layer, a normalization layer, a linear rectification layer, and a 3 × 3 deconvolutional layer are repeatedly added. Finally, a 1 × 1 convolutional layer is added as a full linkage layer, and the averaged outcome is inputted to the regression layer to calculate the error value of the dielectric coefficient distribution.

The cost function is defined as follows:

(10) $$\begin{array}{c} argmin\\ A_{i1},i \end{array}:\overset{N_{t}}{\underset{N=1}{\sum }}f\left(A_{i1}\left(\varepsilon_{z}^{\alpha }\right),\varepsilon_{z}\right)+Q_{1}\left(i\right)$$

(11) $$\begin{array}{c} argmin\\ A_{i2},i \end{array}:\overset{N_{t}}{\underset{N=1}{\sum }}f\left(A_{i2}\left(\begin{array}{c} \varepsilon_{x}^{\alpha }\\ \varepsilon_{y}^{\alpha } \end{array}\right),\left(\begin{array}{c} \varepsilon_{x}\\ \varepsilon_{y} \end{array}\right)\right)+Q_{2}\left(i\right)$$

where $A_{i1}$ and $A_{i2}$ represent the neural network architecture parameters; *f* represents the error; $\varepsilon_{x}^{\alpha }$, $\varepsilon_{y}^{\alpha }$, and $\varepsilon_{z}^{\alpha }$ represent the approximate permittivity coefficients; and $Q_{1}\left(i\right)$ and $Q_{2}\left(i\right)$ represent the regularization functions.

It is well known that CNNs have great advantages in regard to images [76,77]. Therefore, we choose CNN architecture to reconstruct microwave images in this study. The reasons for choosing a CNN include the following:

(1) Inserting a skip connection in the input and output of a CNN can avoid the gradient disappearance problem during the training process.
(2) Adding down-sampling to the CNN contraction network can give a large field-of-view effect to the input image and thus improve the prediction capability of each pixel of the output image.
(3) Adding batch normalization layer to a CNN can alleviate the internal covariance bias. Other than that, it also accelerates the training time and reduces the dependence of the gradient on parameters or initial values.
(4) Designating ReLu as the activation function of a CNN to reduce the interdependence between the parameters can effectively alleviate the occurrence of overfitting.

## 4. Discussion

In our exploration, we assume that anisotropic objects are placed in free space and that the emitters and receivers are placed uniformly around the scatter object. TM and TE waves are emitted in different directions to irradiate the anisotropic objects. We then receive the scattered field information from the unknown scatterers. The received scattered field information is used to estimate the preliminary estimated permittivity by BPS and DCS and is inputted to the CNN to reconstruct an accurate electromagnetic image.

In the simulation environment, we divide the edge constant size of the scatterer into $\frac{0.2\lambda_{0}}{\sqrt{\varepsilon_{r}}}$; $\lambda_{0}$ denotes the wavelength in free space; and $\varepsilon_{r}$ denotes the relative permittivity of the anisotropic object. We define the dielectric coefficient of the scatterer as 1 to 2.5, with the frequency of the incident wave as 3 GHz, and set up 32 transmitter and receiver antennas uniformly. In order to simulate the real environment, we add 5% and 20% Gaussian noise to the simulated environment for investigation. We use the scattered field information to estimate the dielectric coefficient distribution by BPS and DCS to reduce the complexity of the neural network training process. In the artificial intelligence part, we partition the dielectric coefficient distribution estimated from BPS and DCS into 80% for the training set and 20% for the test set. Finally, we use the stochastic gradient descent method to train the neural network. We set the training parameters as momentum of 0.99, learning rate of $10^{-6}$ to $10^{-8}$, and max epoch of 200. We shuffle the training data in each epoch.

We define the root mean square error formula in Equation (12) to evaluate the performance of each scenario.

(12) $$RMSE=\frac{1}{M_{t}}\frac{\sum_{i=1}^{M_{t}}\|{\overset{=}{\varepsilon }}_{r}-{\overset{=}{\varepsilon }}_{r}^{r}\|{}_{F}}{\left\|{\overset{=}{\varepsilon }}_{r}{}_{F}\right\|}$$

${\overset{=}{\varepsilon }}_{r}$ and ${\overset{=}{\varepsilon }}_{r}^{r}$ are the true and reconstructed relative permittivity profiles respectively; $M_{t}$ represents the number of tests conducted, while *F* denotes the Frobenius norm.

Next, we define the structural similarity index measure in Equation (13) for comparing the reconstruction results of each scenario.

(13) $$SSIM=\frac{\left(2\mu_{\tilde{y}}\mu_{y}+C_{1}\right)\left(2\sigma_{\tilde{y}y}+C_{2}\right)}{\left(\mu_{\tilde{y}}^{2}+\mu_{y}^{2}+C_{1}\right)\left(\sigma_{\tilde{y}}^{2}+\sigma_{y}^{2}+C_{2}\right)}$$

Here, $\tilde{y}$ and y are the reconstructed and true relative permittivity profiles, respectively; $\mu_{y}$ is the mean of y; $\sigma_{\tilde{y}}^{2}$ is the variance of y; and $\sigma_{\tilde{y}y}$ is the covariance of $\tilde{y}$ and y. In order to eliminate the zero denominator, two small constraints, $C_{1}={\left(K_{1}D\right)}^{2}$ and $C_{2}={\left(K_{2}D\right)}^{2}$, are added, where $K_{1}=0.01$ and $K_{2}=0.03$ are the two hyperparameters; D indicates the dynamic range of pixels for targeted image y [73].

### 4.1. Relative Permittivity between 1 and 1.5

In this case, we set the permittivity distribution between 1 and 1.5. We assume 10 scatterers with different permittivity distributions, which can move arbitrarily at 50 different positions within the measurement area. We add 20% Gaussian noise to the simulated environment. Then, we estimate the dielectric coefficient distribution from the scattered field information by BPS and DCS and input it to the CNN for electromagnetic image reconstruction. Finally, we compare the reconstruction results of two different input data.

**Figure 3:**
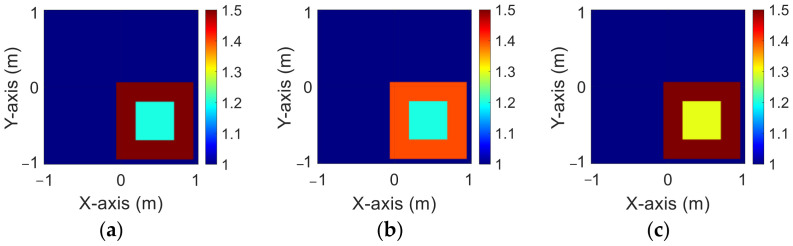
Ground truth with relative permittivity 1 to 1.5 (**a**) $\varepsilon_{z}$; (**b**) $\varepsilon_{x}$; (**c**) $\varepsilon_{y}$.

**Figure 4:**
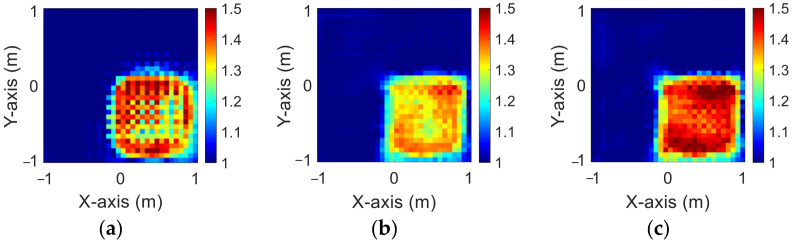
Reconstructed images with relative permittivity 1 to 1.5 at 20% noise by BPS (**a**) $\varepsilon_{z}$; (**b**) $\varepsilon_{x}$; (**c**) $\varepsilon_{y}$.

**Figure 5:**
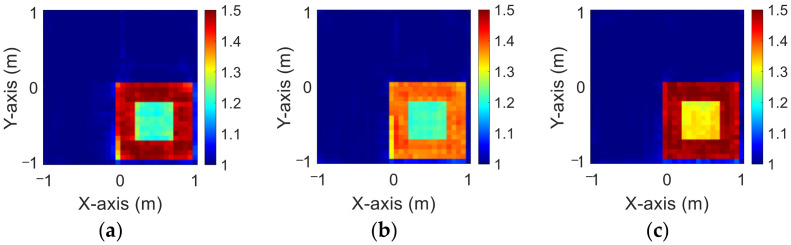
Reconstructed images with relative permittivity 1 to 1.5 at 20% noise by DCS (**a**) $\varepsilon_{z}$; (**b**) $\varepsilon_{x}$; (**c**) $\varepsilon_{y}$.

Figure 3a–c show the original permittivity distributions of $\varepsilon_{z}$, $\varepsilon_{x}$, and $\varepsilon_{y}$ scatterers, respectively. Figure 4a–c show, respectively, the reconstructed permittivity distributions of $\varepsilon_{z}$, $\varepsilon_{x}$, and $\varepsilon_{y}$ by the CNN for BPS input. Figure 5a–c show, respectively, the reconstructed permittivity distributions of $\varepsilon_{z}$, $\varepsilon_{x}$, and $\varepsilon_{y}$ by the CNN for DCS input. Figure 4 illustrates that BPS can only reconstruct the position of the scatterer and the coarse distribution of the permittivity. In the TM case, the error rate of the BPS reconstruction result is 7.9%, and the similarity is 78.3%. In the TE case, the error rate of the BPS reconstruction result for $\varepsilon_{x}$ and $\varepsilon_{y}$ are 5.6% and 6.4%, respectively, while the similarity of the BPS reconstruction result for $\varepsilon_{x}$ and $\varepsilon_{y}$ are 84.3% and 84.5%, respectively. Figure 5 demonstrates that DCS can accurately reconstruct the scatterer’s position and dielectric coefficient distribution. In the TM case, the error rate of the DCS reconstruction result is 3.1%, and the similarity is 89.9%. In the TE case, the error rate of the DCS reconstruction result for $\varepsilon_{x}$ and $\varepsilon_{y}$ are 2.1% and 2%, respectively. And the similarity of the BPS reconstruction result for $\varepsilon_{x}$ and $\varepsilon_{y}$ are 93.2% and 94.1%, respectively. The results conclude that both DCS and BPS are able to reconstruct their shapes and sizes in the 1–1.5 scheme and that DCS is also able to reconstruct the exact dielectric coefficient distribution. The RMSE and SSIM of the reconstruction results are listed in Table 1. We can see that reconstructed images by DCS are better than those reconstructed by BPS in terms of both error rate and similarity.

### 4.2. Relative Permittivity between 1.5 and 2

In this case, we set the permittivity distribution between 1.5 and 2. We assume 10 scatterers with different permittivity distributions, which can move arbitrarily at 50 different positions within the measurement area. We add 5% Gaussian noise to the simulated environment. Then, we estimate the dielectric coefficient distribution from the scattered field information by BPS and DCS and input it to the CNN for electromagnetic image reconstruction. Finally, we compare the reconstruction results of two different input data.

**Figure 6:**
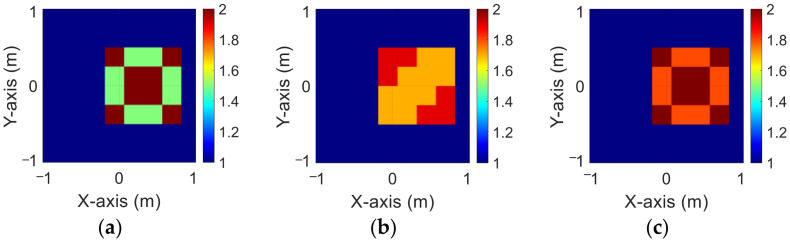
Ground truth with relative permittivity 1.5 to 2 (**a**) $\varepsilon_{z}$; (**b**) $\varepsilon_{x}$; (**c**) $\varepsilon_{y}$.

**Figure 7:**
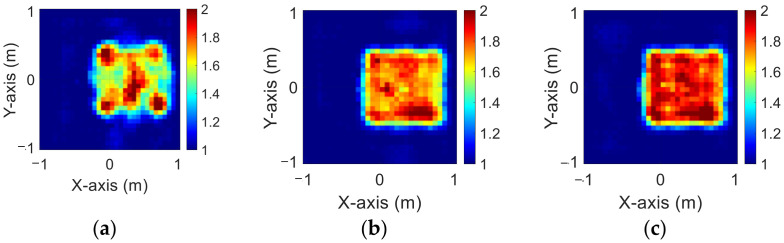
Reconstructed images with relative permittivity 1.5 to 2 at 5% noise by BPS (**a**) $\varepsilon_{z}$; (**b**) $\varepsilon_{x}$; (**c**) $\varepsilon_{y}$.

**Figure 8:**
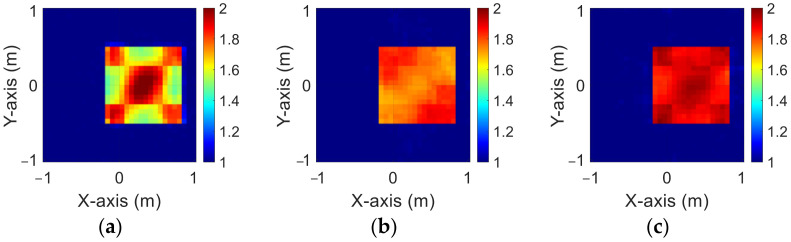
Reconstructed images with relative permittivity 1.5 to 2 at 5% noise by DCS (**a**) $\varepsilon_{z}$; (**b**) $\varepsilon_{x}$; (**c**) $\varepsilon_{y}$.

Figure 6a–c show the original permittivity distributions of $\varepsilon_{z}$, $\varepsilon_{x}$, and $\varepsilon_{y}$ scatterers, respectively. Figure 7a–c show, respectively, the reconstructed permittivity distributions of $\varepsilon_{z}$, $\varepsilon_{x}$, and $\varepsilon_{y}$ by the CNN for BPS input. Figure 8a–c show, respectively, the reconstructed permittivity distributions of $\varepsilon_{z}$, $\varepsilon_{x}$, and $\varepsilon_{y}$ by the CNN for DCS input. Figure 7 illustrates that BPS is capable of reconstructing the ambiguous scatterer position and dielectric coefficient distribution. In the TM case, the error rate of the BPS reconstruction result is 11.5%, and the similarity is 81.5%. In the TE case, the error rate of the BPS reconstruction result for $\varepsilon_{x}$ and $\varepsilon_{y}$ are 9.1% and 9.8%, respectively, while the similarity of the BPS reconstruction result for $\varepsilon_{x}$ and $\varepsilon_{y}$ are 85.8% and 85.5%, respectively. Figure 8 demonstrates that DCS can not only reconstruct the position of the scatterer but also the ambiguous distribution of the dielectric coefficient. In the TM case, the error rate of the DCS reconstruction result is 6.6%, and the similarity is 80%. In the TE case, the error rate of the DCS reconstruction result for $\varepsilon_{x}$ and $\varepsilon_{y}$ are 3.9% and 3.8%, respectively. And the similarity of the BPS reconstruction result for $\varepsilon_{x}$ and $\varepsilon_{y}$ are 85.6% and 86.1%, respectively. The results conclude that both DCS and BPS are able to reconstruct their shapes and sizes in the 1.5–2 scheme, and that DCS also overwhelms BPS in reconstructing more accurate permittivity distributions. The RMSE and SSIM of the reconstruction results are listed in Table 2. We can see that reconstructed images by DCS are better than those reconstructed by BPS in terms of both error rate and similarity.

### 4.3. Relative Permittivity between 2 and 2.5

The modified National Institute of Standards and Technology database (MNIST) is a large handwritten dataset that was built by the National Institute of Standards and Technology in 1988. The MNIST dataset has been widely used in machine learning and deep learning for image recognition. The dataset has 70,000 images of handwritten numbers from 0–9, where each image is in a grayscale of 28 × 28 pixels. In this case, we selected MINST and set its dielectric distribution between 2 and 2.5. In our simulation environment, we select 50 images randomly as the data set and add 5% Gaussian noise. Then, we estimate the dielectric coefficient distribution from the scattered field information by BPS and DCS and input it to the CNN for electromagnetic image reconstruction. Finally, we compare the reconstruction results of two different input data.

**Figure 9:**
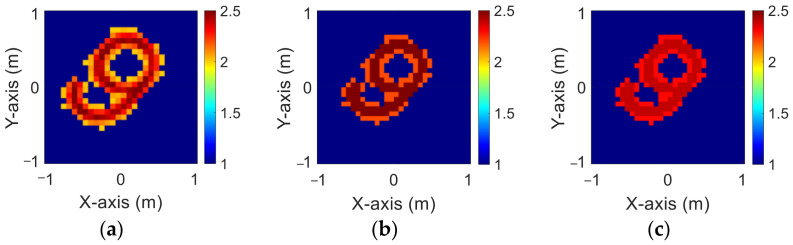
Ground truth with relative permittivity 2 to 2.5 (**a**) $\varepsilon_{z}$; (**b**) $\varepsilon_{x}$; (**c**) $\varepsilon_{y}$.

**Figure 10:**
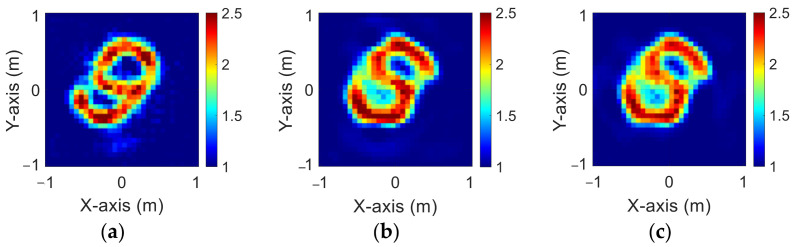
Reconstructed image with relative permittivity 2 to 2.5 at 5% noise by BPS (**a**) $\varepsilon_{z}$; (**b**) $\varepsilon_{x}$; (**c**) $\varepsilon_{y}$.

**Figure 11:**
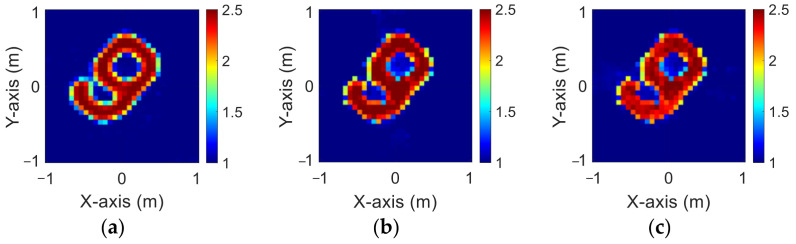
Reconstructed image with relative permittivity 2 to 2.5 at 5% noise by DCS (**a**) $\varepsilon_{z}$; (**b**) $\varepsilon_{x}$; (**c**) $\varepsilon_{y}$.

Figure 9a–c show the original permittivity distributions of $\varepsilon_{z}$, $\varepsilon_{x}$, and $\varepsilon_{y}$ scatterers, respectively. Figure 10a–c show, respectively, the reconstructed permittivity distributions of $\varepsilon_{z}$, $\varepsilon_{x}$, and $\varepsilon_{y}$ by the CNN for BPS input. Figure 11a–c show, respectively, the reconstructed permittivity distributions of $\varepsilon_{z}$, $\varepsilon_{x}$, and $\varepsilon_{y}$ by the CNN for DCS input. The RMSE and SSIM of the reconstructed results are listed in Table 3. In summary, Figure 10 shows that BPS can reconstruct the outline and dielectric coefficient distribution of the relatively faint handwritten digit 9. In the TM case, the error rate of the BPS reconstruction result is 13.2%, and the similarity is 73.5%. In the TE case, the error rate of the BPS reconstruction result for $\varepsilon_{x}$ and $\varepsilon_{y}$ are 20.9% and 21.1%, respectively, while the similarity of the BPS reconstruction result for $\varepsilon_{x}$ and $\varepsilon_{y}$ are 65.9% and 64.8%, respectively. Figure 11 demonstrates that DCS is able to reconstruct an accurate handwritten digit 9 as well as their dielectric coefficient distributions. In the TM case, the error rate of the BPS reconstruction result is 10.3%, and the similarity is 75.5%. In the TE case, the error rate of the DCS reconstruction result for $\varepsilon_{x}$ and $\varepsilon_{y}$ are 13.1% and 10.6%, respectively. And the similarity of the DCS reconstruction result for $\varepsilon_{x}$ and $\varepsilon_{y}$ are 76.2% and 79.9%, respectively. The results conclude that both DCS and BPS are able to reconstruct the shape and size of the handwritten digit. Meanwhile, we also discover that the reconstructed results are more ambiguous, and the dielectric coefficient distribution is less accurate for the BPS scheme. However, DCS is able to reconstruct an accurate dielectric coefficient distribution and high-resolution images. Numerical results display that DCS performs better than BPS in terms of error rate and similarity in image reconstruction.

### 4.4. Different Levels of Noise

In this section, we use the trained model with a 5% noise level to predict the 10% and 20% noise test data.

**Figure 12:**
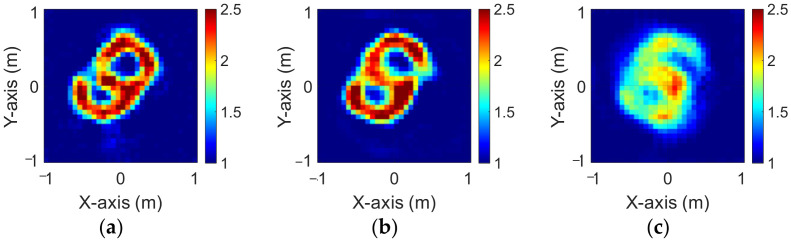
Reconstructed images with 10% noise by BPS (**a**) $\varepsilon_{z}$; (**b**) $\varepsilon_{x}$; (**c**) $\varepsilon_{y}$.

**Figure 13:**
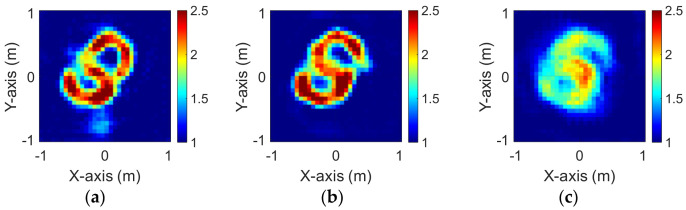
Reconstructed images with 20% noise by BPS (**a**) $\varepsilon_{z}$; (**b**) $\varepsilon_{x}$; (**c**) $\varepsilon_{y}$.

**Figure 14:**
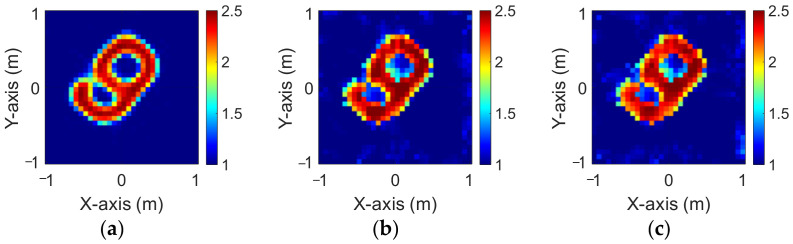
Reconstructed images with 10% noise by DCS (**a**) $\varepsilon_{z}$; (**b**) $\varepsilon_{x}$; (**c**) $\varepsilon_{y}$.

**Figure 15:**
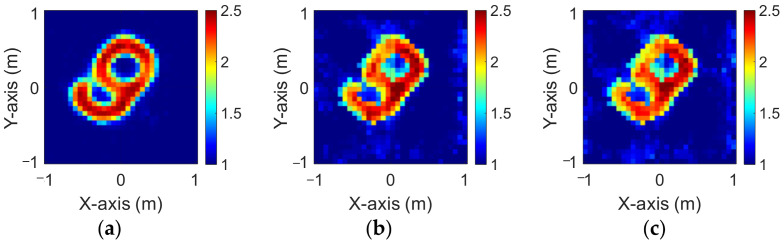
Reconstructed images with 20% noise by DCS (**a**) $\varepsilon_{z}$; (**b**) $\varepsilon_{x}$; (**c**) $\varepsilon_{y}$.

Figure 12a–c and Figure 13a–c show the reconstruction results of BPS under 10% and 20% noise levels, respectively. Figure 14a–c and Figure 15a–c show the reconstruction results by DCS under 10% and 20% noise levels, respectively. The RMSE and SSIM of the reconstructed results for 10% and 20% noise levels are listed in Table 4 and Table 5, respectively. Results show that the reconstruction results are blurred for larger noise levels in both DCS and BPS.

### 4.5. Experimental Dataset

**Figure 16:**
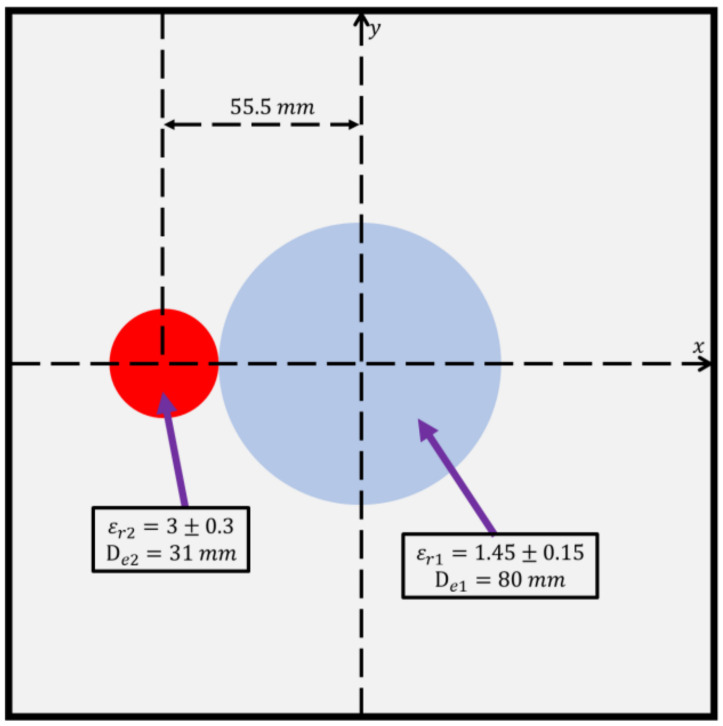
The FoamDielExt schematic diagram.

In this case, we use the experimental dataset provided by the Fresnel Institute [77]. This is to verify the effectiveness of our proposed BPS and DCS for TM and TE schemes. The experimental dataset is set up with eight transmitters and 241 receivers in the environment. The distance between the transmitters and the measured objects is 1.67 m. Since the transmitters in this dataset use horn antennas to measure the scattered fields, no transmitters are placed on either side of the receivers. We select the measured data of FoamDielExt in the TM and TE cases. The FoamDielExt pair consists of a large (SAITEC SBF 300) and a small (Berlon) cylinder. The large cylinder with a diameter of 80 mm has a permittivity of $\varepsilon_{r}$ = 1.45 ± 0.15, and the small cylinder with a diameter of 31 mm has a permittivity of $\varepsilon_{r}$ = 3 ± 0.3. In our simulation environment, we place the scatterers in a 320 × 320 mm measurement space and TM and TE incident waves with a frequency of 3 GHz, respectively. Then, we use the received scattered fields for calibration. During the calibration process, we select the simulated scattered fields received on the other side of the incident angle for normalization. The FoamDielExt schematic diagram is shown in Figure 16.

**Figure 17:**
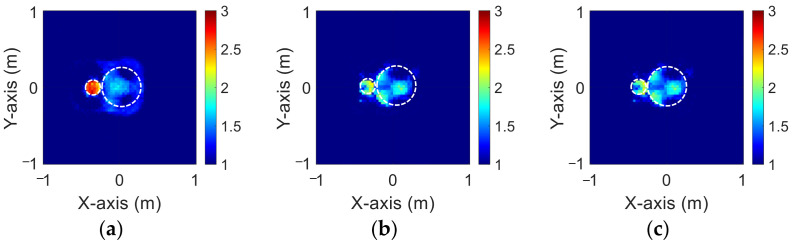
Reconstructed experimental image by BPS (**a**) $\varepsilon_{z}$; (**b**) $\varepsilon_{x}$; (**c**) $\varepsilon_{y}$.

**Figure 18:**
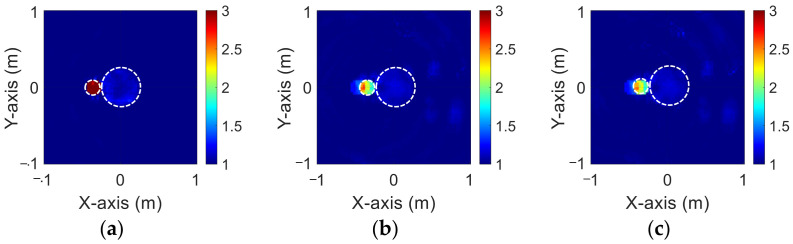
Reconstructed experimental image by DCS (**a**) $\varepsilon_{z}$; (**b**) $\varepsilon_{x}$; (**c**) $\varepsilon_{y}$.

Figure 17a–c show, respectively, the reconstructed permittivity distributions of $\varepsilon_{z}$, $\varepsilon_{x}$, and $\varepsilon_{y}$ by the CNN for BPS input, respectively. Figure 18a–c show, respectively, the reconstructed permittivity distributions of $\varepsilon_{z}$, $\varepsilon_{x}$, and $\varepsilon_{y}$ by the CNN for DCS input. The RMSE and SSIM of the reconstructed results are listed in Table 6. In our investigation, Figure 16 shows that BPS can reconstruct the coarse scatterer location and dielectric coefficient distribution. In the TM case, the error rate of the BPS reconstruction result is 13.7%, and the similarity is 83.2%, while in the TE case, the error rate of the BPS reconstruction result for $\varepsilon_{x}$ and $\varepsilon_{y}$ are 11.5% and 11.6%, respectively. And the similarity of the BPS reconstruction result for $\varepsilon_{x}$ and $\varepsilon_{y}$ are 90.5% and 90.4%, respectively. Figure 18 demonstrates that DCS is able to reconstruct the exact object location and the relative dielectric coefficient distribution. In the TM case, the error rate of the BPS reconstruction result is 10.5%, and the similarity is 88.5%. In the TE case, the error rate of the DCS reconstruction result for $\varepsilon_{x}$ and $\varepsilon_{y}$ are 11.5% and 11.6%, respectively. And the similarity of the DCS reconstruction result for $\varepsilon_{x}$ and $\varepsilon_{y}$ are 81.4% and 81.1%, respectively. The results conclude that both DCS and BPS are able to reconstruct the shape and size of the experimental data. Meanwhile, we also discover that the reconstructed results are more ambiguous, and the dielectric coefficient distribution is less accurate for the BPS scheme. However, DCS is able to reconstruct an accurate dielectric coefficient distribution and high-resolution images. Numerical results display that DCS performs better than BPS in terms of error rate and similarity in image reconstruction.

## 5. Conclusions

In this paper, we investigate the electromagnetic backscattering of TM and TE incident waves from anisotropic objects. In other words, we use TM and TE incident anisotropic objects to estimate the preliminary dielectric coefficients from the received scattered field information by BPS and DCS. We believe this is an efficient scheme that could simplify the training process of neural networks. Eventually, the estimated dielectric coefficients are inputted to a CNN for image reconstruction. The main contribution of this paper is that since biaxial anisotropic scatterers have different components of dielectric coefficients along different transverse directions, the problem will be more complicated than the purely quantitative TM polarization case. Numerical results show that DCS can effectively and accurately reconstruct the dielectric coefficient distribution of anisotropic objects regardless of the noise and training parameters, while BPS can only reconstruct a blurred image. There is still room for the development of artificial intelligence technology in electromagnetic imaging. In the future, we will further investigate the study of anisotropic buried dielectric objects, which will be our next research goal. The use of different neural network architectures with different loss functions will also be one of our future research focuses.

## Figures and Tables

**Table 1:** RMSE and SSIM for permittivity between 1 and 1.5 with 20% noise.

Reconstruction Performance		$\varepsilon_{z}$	$\varepsilon_{x}$	$\varepsilon_{y}$
BPS	RMSE	7.9%	5.6%	6.4%
	SSIM	78.3%	84.3%	84.5%
DCS	RMSE	3.1%	2.1%	2%
	SSIM	89.9%	93.2%	94.1%

**Table 2:** RMSE and SSIM for permittivity between 1.5 and 2 with 5% noise.

Reconstruction Performance		$\varepsilon_{z}$	$\varepsilon_{x}$	$\varepsilon_{y}$
BPS	RMSE	11.5%	9.1%	9.8%
	SSIM	81.5%	85.8%	85.5%
DCS	RMSE	6.6%	3.9%	3.8%
	SSIM	80%	85.6%	86.1%

**Table 3:** RMSE and SSIM for permittivity between 2 and 2.5 with 5% noise.

Reconstruction Performance		$\varepsilon_{z}$	$\varepsilon_{x}$	$\varepsilon_{y}$
BPS	RMSE	13.2%	20.9%	21.1%
	SSIM	76.5%	65.9%	64.8%
DCS	RMSE	10.3%	13.1%	10.6%
	SSIM	75.5%	76.2%	79.9%

**Table 4:** RMSE and SSIM for 10% noise.

Reconstruction Performance		$\varepsilon_{z}$	$\varepsilon_{x}$	$\varepsilon_{y}$
BPS	RMSE	14.3%	38.4%	37.9%
	SSIM	75%	42.1%	42.1%
DCS	RMSE	14.3%	32.6%	28.3%
	SSIM	71%	40.3%	34.5%

**Table 5:** RMSE and SSIM for 20% noise.

Reconstruction Performance		$\varepsilon_{z}$	$\varepsilon_{x}$	$\varepsilon_{y}$
BPS	RMSE	16.1%	39%	38.6%
	SSIM	71%	37%	36.9%
DCS	RMSE	17.4%	40.3%	34.5%
	SSIM	60.6%	29.8%	31.6%

**Table 6:** RMSE and SSIM for experimental dataset.

Reconstruction Performance		$\varepsilon_{z}$	$\varepsilon_{x}$	$\varepsilon_{y}$
BPS	RMSE	13.7%	11.5%	11.6%
	SSIM	83.2%	90.5%	90.4%
DCS	RMSE	10.5%	11.5%	11.6%
	SSIM	88.5%	81.4%	81.1%

## Data Availability

Not applicable.
